# Low intake of β carotene and dietary fiber from vegetables and fruits in patients with chronic kidney disease

**DOI:** 10.1038/s41598-022-24471-4

**Published:** 2022-11-19

**Authors:** Toshiaki Nakano, Shigeru Tanaka, Kazuhiko Tsuruya, Takanari Kitazono

**Affiliations:** 1 Center for Cohort Studies, Kyushu University, 3-1-1 Maidashi, Higashi-Ku, Fukuoka, 812-8582 Japan; 2 Department of Medicine and Clinical Science, Graduate School of Medical Sciences, Kyushu University, 3-1-1 Maidashi, Higashi-Ku, Fukuoka, 812-8582 Japan; 3 Department of Nephrology, Nara Medical University, Kashihara, Nara Japan

**Keywords:** Kidney diseases, Epidemiology, Chronic kidney disease

## Abstract

Patients with chronic kidney disease (CKD) occasionally need to restrict their consumption of vegetables and fruits. However, recent evidence suggests that plant-based diets have beneficial effects in patients with CKD. We aimed to determine the sufficiency of β carotene and dietary fiber intake in patients with CKD. We conducted a cross-sectional study among 4476 patients registered in the Fukuoka Kidney Disease Registry (FKR) study, a Japanese prospective cohort study of patients with CKD. Data from 3545 patients were analyzed after excluding cases with insufficient information. We evaluated the relationship between CKD stages and the intake of vegetables and fruits. The intake of β carotene and dietary fiber in CKD stages was evaluated using analysis of covariance. As the CKD stage advanced, the intake of vegetables, green leafy vegetables, and fruits significantly decreased (P-value for all trends < 0.01). The intake of vegetables significantly decreased as the CKD stage advanced (P for trend < 0.01). After adjusting for confounding factors, the intake of β carotene and dietary fiber also decreased (both P < 0.01) as the CKD stage advanced. Patients with CKD had insufficient vegetable and fruit intake and a lack of β carotene and dietary fiber from vegetables and fruits.

## Introduction

Hyperkalemia is associated with arrhythmia and sudden death and increases mortality in patients with chronic kidney disease (CKD)^1, 2^. To prevent hyperkalemia, the 2020 update of the clinical practice guideline for nutrition in CKD provided by the National Kidney Foundation’s Kidney Disease Outcomes Quality Initiative indicates that “it is reasonable to adjust dietary potassium intake to maintain serum potassium within the normal range”^3^. The dietary therapy for patients with CKD occasionally needs to restrict the consumption of vegetables and fruits. However, a healthy diet that includes consuming fruits and vegetables several times a day is widely recommended for preventing cardiovascular disease (CVD), diabetes, and cancer^4^. Nevertheless, the percentage of the adult population that meets the fruit and vegetable intake recommendation is low even in healthy adults^5^. In 2015, 12.2% of adults met the fruit intake recommendation and 9.3% met the vegetable intake recommendation in the United States^5^. A lower proportion of adults with CKD may meet fruit and vegetable intake recommendations compared with healthy patients; however, information on fruit and vegetable intake in CKD patients is lacking. Recent studies suggest that a diet rich in vegetables and fruits was associated with a lower risk of mortality in patients with CKD and hemodialysis patients^6, 7^. Whereas animal-based protein ingestion promotes an acidic environment, inflammation, and renal hyperfiltration, a plant-based diet produces an alkaline environment, is anti-inflammatory, and contains reno-protective properties^8^.

Retinoic acid is the major metabolite of vitamin A and promotes embryonic development, post-natal growth, vision, epithelial differentiation, and immunity^9^. β carotene is provitamin A, which can be transformed into retinoic acid in the body^10^. β carotene can be found in green leafy vegetables and orange and yellow fruits and is reported to have antioxidant effects^10^. Moreover, dietary fiber has numerous physiological functions and reduces uremic toxins^8^. Whether the intake of β carotene and dietary fiber is sufficient in patients with CKD is unknown.

In the present study, we aimed to investigate the relationship between CKD stages and the intake of vegetables, fruits, β carotene and dietary fiber.

## Results

### Study participants and baseline characteristics

The clinical characteristics of participants at each CKD stage are shown in Table 1. Advanced CKD stages were positively associated with age, male sex, the presence of diabetes, a history of CVD, high systolic blood pressure, high diastolic blood pressure, high C-reactive protein concentrations, a high urinary protein/creatinine ratio, but were negatively associated with serum albumin and low-density lipoprotein (LDL) cholesterol concentrations.

**Table 1:** Baseline characteristics according to CKD stage.

CKD stage	G1	G2	G3a	G3b	G4	G5	P
Number	161	637	698	770	873	406	
Age, years	36 (27–49)	59 (46–68)	66 (56–73)	70 (63–77)	73 (65–80)	72 (64–79)	< 0.01
Male, %	36	43	55	65	62	59	< 0.01
Diabetes, %	2	1	5	8	16	29	< 0.01
History of cardiovascular disease, %	1	7	11	22	31	30	< 0.01
Systemic blood pressure, mmHg	117 (109–128)	126 (116–138)	130 (119–140)	130 (120–142)	132 (121–144)	135 (124–146)	< 0.01
Diastolic blood pressure, mmHg	70 (63–79)	75 (68–83)	76 (70–84)	74 (68–82)	73 (65–81)	72 (65–80)	< 0.01
Body mass index, kg/m^2^	21.2 (19.3–24.4)	22.9 (20.5–25.9)	23.2 (21.0–25.9)	23.1 (21.0–25.6)	22.7 (20.5–25.3)	22.7 (20.2–25.0)	0.18
Albumin, g/dL	4.3 (4.0–4.5)	4.2 (4.0–4.5)	4.2 (4.0–4.4)	4.1 (4.0–4.4)	4.0 (3.7–4.2)	3.9 (3.6–4.1)	< 0.01
LDL cholesterol, mg/dL	103 (88–129)	110 (94–128)	108 (88–129)	105 (86–125)	100 (79–122)	92 (71–116)	< 0.01
C-reactive protein, mg/dL	0.03 (0.01–0.08)	0.04 (0.02–0.09)	0.05 (0.02–0.11)	0.06 (0.03–0.13)	0.06 (0.03–0.14)	0.06 (0.03–0.18)	< 0.01
Urinary protein/creatinine, g/gCr	0.11 (0.05–0.31)	0.16 (0.07–0.56)	0.17 (0.07–0.56)	0.28 (0.10–0.98)	0.66 (0.27–1.87)	1.75 (0.89–3.39)	< 0.01

CKD, chronic kidney disease; Cr, creatinine; LDL, low-density lipoprotein; s.e., standard error .

### Relationship between chronic kidney disease stage and the intake of vegetables and fruits

We evaluated the relationship between CKD stage and the intake of vegetables, green leafy vegetables, and fruits. As the CKD stage advanced, the intake of vegetables, green leafy vegetables, and fruits significantly decreased (P-values for all trends < 0.01; Tables 2, 3, 4). After adjusting for confounding factors, the trends for the intake of vegetables, green leafy vegetables, and fruits significantly decreased as the CKD stage advanced (P-values for all trends < 0.01; Tables 2, 3, 4). The intake of green leafy vegetables and fruits decreased significantly, especially in CKD G5 patients. The Japanese Ministry of Health, Labour and Welfare recommends a target of 350 g of vegetables per day; only 23.1% of participants achieved this target intake in our study (18.0%, 25.3%, 29.2%, 24.3%, 21.0%, and 13.8%%, in CKDG1, G2, G3a, G3b, G4, and G5, respectively). To evaluate whether CKD patients consume a sufficient amount of vegetables, we evaluated the relationship between CKD stage and the prevalence of an intake of less than 350 g of vegetables per day (Table 5). As the CKD stage advanced, the prevalence of a low intake (< 350 g) of vegetables significantly increased (P < 0.01).

**Table 2:** Association between CKD stage and the quantity of vegetables consumed per day.

CKD stage	Unadjusted		P for trend	Age- and sex-adjusted		P for trend	Multivariate-adjusted^a^		P for trend
	Mean	s.e.		Mean	s.e.		Mean	s.e.	
CKD G1	259.0	23.7	< 0.01	263.9	25.7	< 0.01	271.0	27.1	< 0.01
CKD G2	282.4	11.9		278.6	12.3		281.5	13.0	
CKD G3a	307.6	11.4		307.6	11.3		307.7	11.9	
CKD G3b	274.5	10.8		278.4	10.9		283.4	11.4	
CKD G4	240.2	10.2		239.6	10.4		245.3	11.1	
CKD G5	196.1**	14.9		193.9*	14.9		197.4	16.6	

CKD, chronic kidney disease; s.e., standard error.

**P < 0.01 vs mean value of CKD G1, *P < 0.05 vs mean value of CKD G1.

^a^Adjusted for age, sex, diabetes, history of cardiovascular disease, systolic blood pressure, body mass index, serum albumin concentration, low-density lipoprotein cholesterol, log-C-reactive protein, and urinary protein/creatinine ratio.

**Table 3:** Association between CKD stage and the quantity of green leafy vegetables consumed per day.

CKD stage	Unadjusted		P for trend	Age- and sex-adjusted		P for trend	Multivariate-adjusted^a^		P for trend
	Mean	s.e.		Mean	s.e		Mean	s.e.	
CKD G1	111.7	10.6	< 0.01	113.7	11.4	< 0.01	117.5	12.1	< 0.01
CKD G2	121.2	5.3		119.2	5.4		121.5	5.8	
CKD G3a	126.3	5.1		126.3	5.0		127.2	5.3	
CKD G3b	111.3	4.8		113.4	4.8		116.1	5.1	
CKD G4	95.0	4.5		94.8	4.6		97.0	5.0	
CKD G5	79.5**	6.6		78.4**	6.6		79.0*	7.4	

CKD, chronic kidney disease; s.e., standard error.

**P < 0.01 vs mean value of CKD G1, *P < 0.05 vs mean value of CKD G1.

^a^Adjusted for age, sex, diabetes, history of cardiovascular disease, systolic blood pressure, body mass index, serum albumin concentration, low-density lipoprotein cholesterol, log-C-reactive protein, and urinary protein/creatinine ratio.

**Table 4:** Association between CKD stage and the quantity of fruits consumed per day.

CKD stage	Unadjusted		P for trend	Age- and sex-adjusted		P for trend	Multivariate-adjusted^a^		P for trend
	Mean	s.e.		Mean	s.e.		Mean	s.e.	
CKD G1	184.3	28.7	< 0.01	228.7	31.1	< 0.01	235.6	32.9	< 0.01
CKD G2	248.8	14.4		257.5	14.9		256.3	15.7	
CKD G3a	248.0	13.8		250.3	13.7		247.2	14.4	
CKD G3b	222.3	13.1		220.4	13.2		224.7	13.8	
CKD G4	180.0	12.3		169.5	12.6		172.9	13.5	
CKD G5	121.6	18.1		111.7**	18.1		125.4**	20.2	

CKD, chronic kidney disease; s.e., standard error.

**P < 0.01 vs mean value of CKD G1.

^a^Adjusted for age, sex, diabetes, history of cardiovascular disease, systolic blood pressure, body mass index, serum albumin concentration, low-density lipoprotein cholesterol, log-C-reactive protein, and urinary protein/creatinine ratio.

**Table 5:** Association between CKD stage and the prevalence of low intake of vegetables.

CKD stage	No. of events/patients	Age- and sex-adjusted			Multivariable-adjusted^a^		
		OR (95% CI)	P value	P for trend	OR (95% CI)	P value	P for trend
CKD G1	132/161	1.00 (reference)		< 0.01	1.00 (reference)		< 0.01
CKD G2	476/637	0.80 (0.50–1.26)	0.33		0.83 (0.52–1.32)	0.42	
CKD G3a	494/698	0.66 (0.42–1.06)	0.08		0.71 (0.44–1.14)	0.16	
CKD G3b	583/770	0.87 (0.54–1.40)	0.56		0.88 (0.54–1.43)	0.61	
CKD G4	690/873	1.12 (0.69–1.81)	0.65		1.10 (0.67–1.81)	0.72	
CKD G5	350/406	1.88 (1.11–3.21)	0.02		1.85 (1.06–3.24)	0.03	

CKD, chronic kidney disease; CI, confidence interval; OR, odds ratio; s.e., standard error.

^a^Adjusted for age, sex, diabetes, history of cardiovascular disease, systolic blood pressure, body mass index, serum albumin concentration, low-density lipoprotein cholesterol, log-C-reactive protein, and urinary protein/creatinine ratio.

### Relationship between CKD stage and the intake of β carotene and dietary fiber

Beta carotene is reported to have an antioxidant effect and retinoic acid metabolized from β carotene plays a role in renal protection^11, 12^. We calculated the β carotene intake from vegetables and fruits and evaluated the relationship between CKD stage and β carotene intake. As the CKD stage advanced, the β carotene intake trend significantly decreased (P < 0.01; Table 6). This trend was unchanged after adjusting for confounding factors (P < 0.01; Table 6). Moreover, the increased intake of dietary fiber shifts gut microbiota toward a reduced production of uremic toxins^13^. We evaluated the intake of dietary fiber from vegetables and fruits in patients with CKD. As the CKD stage advanced, the dietary fiber intake trend decreased (P < 0.01; Table 7); this trend was unchanged after adjusting for confounding factors (P < 0.01; Table 7).

**Table 6:** Association between CKD stage and amount of β carotene consumed per day.

CKD stage	Unadjusted		P for trend	Age- and sex-adjusted		P for trend	Multivariate-adjusted^a^		P for trend
	Mean	s.e.		Mean	s.e.		Mean	s.e.	
CKD G1	3727	377	< 0.01	3813	408	< 0.01	3879	433	< 0.01
CKD G2	4200	190		4145	196		4214	207	
CKD G3a	4194	181		4195	180		4212	189	
CKD G3b	3686	172		3745	173		3829	182	
CKD G4	3489	162		3477	165		3547	178	
CKD G5	2983**	237		2947*	238		2979*	266	

CKD, chronic kidney disease; s.e., standard error.

**P < 0.01 vs mean value of CKD G1, *P < 0.05 vs mean value of CKD G1.

^a^Adjusted for age, sex, diabetes, history of cardiovascular disease, systolic blood pressure, body mass index, serum albumin concentration, low-density lipoprotein cholesterol, log-C-reactive protein, and urinary protein/creatinine ratio.

**Table 7:** Association between CKD stage and amount of dietary fiber only from fruits and vegetables consumed per day.

CKD stage	Unadjusted		P for trend	Age- and sex-adjusted		P for trend	Multivariate-adjusted^a^		P for trend
	Mean	s.e.		Mean	s.e.		Mean	s.e.	
CKD G1	7.46	0.72	< 0.01	8.14	0.78	<0.01	8.34	0.82	< 0.01
CKD G2	8.73	0.36		8.77	0.37		8.83	0.39	
CKD G3a	9.21	0.34		9.24	0.34		9.20	0.36	
CKD G3b	8.21	0.33		8.26	0.33		8.40	0.34	
CKD G4	7.03	0.31		6.89	0.31		7.04	0.34	
CKD G5	5.54**	0.45		5.37**	0.45		5.58**	0.50	

CKD, chronic kidney disease; s.e., standard error.

**P < 0.01 vs mean value of CKD G1.

^a^Adjusted for age, sex, diabetes, history of cardiovascular disease, systolic blood pressure, body mass index, serum albumin concentration, low-density lipoprotein cholesterol, log-C-reactive protein, and urinary protein/creatinine ratio.

## Discussion

This study examined the relationship between kidney function and the intake of vegetables, fruits, β carotene, and dietary fiber in 3545 patients in the FKR study. Advanced CKD stages were significantly associated with low intakes of vegetables and fruits. The odds ratio of the insufficiency of vegetable consumption significantly increased as the CKD stage advanced (P < 0.01). The intake of β carotene and dietary fiber was significantly lower in advanced CKD patients than in less advanced CKD patients (P for trend < 0.01). In this study, we demonstrated an insufficiency in the quantities of vegetables and fruits and the amount of β carotene and dietary fiber ingested by advanced CKD patients.

Nephrologists have traditionally recommended a limited intake of vegetables and fruits to their renal patients to avoid hyperkalemia in these patients. The Japanese Society of Nephrology’s 2014 dietary recommendations for chronic kidney disease propose restricting potassium intake to 2000 mg per day in CKD G3b patients and 1500 mg per day in CKD G4 and G5 patients^14^. Nephrologists and nutritionists provide CKD patients with dietary instructions to achieve this target intake. Consequently, CKD patients sometimes restrict their consumption of fruits and fresh vegetables. The 2019 report from the Japanese Ministry of Health, Labour and Welfare indicates that the mean intake of vegetables in the general population was 280 g per day. In our study, the mean intake of vegetables in CKD patients was 264.3 g per day. Notably, the mean intake of vegetables in patients with CKD G5 was 196.1 g per day—lower than that in the general population. This result suggests that patients with advanced CKD may be deficient in the nutrients provided by vegetables.

However, emerging evidence suggests that an elevated consumption of vegetables and fruits is associated with lower mortality among adults with mild to moderate CKD and hemodialysis patients^6, 7^. Previous studies of the Mediterranean diet, the Dietary Approaches to Stop Hypertension diet, and healthy plant-based diets have consistently shown the association of these types of diet with lower CKD prevalence or incidence compared with the average diet^15, 16^. The evidence in CKD patients supports that these diets delay the progression of end-stage kidney disease^6, 17^.

Recent evidence suggests that plant-based diets could have additional beneficial effects in patients with CKD. Increased fiber intake improves gut microbiota by reducing the production of uremic toxins^13^. A high intake of fiber also promotes saccharolytic fermentation and increases short-chain fatty acid production^8^. Plant-based diets tend to lower cholesterol levels and body weight^13^. A low intake of vegetables and fruits increases dietary acid load^18^. In contrast to a plant-based diet, a diet that is rich in animal proteins leads to the proliferation of proteolytic bacteria and increases uremic toxins^13^. The uremic milieu in CKD patients leads to disturbances in the intestinal barrier and promotes inflammation, oxidative stress, and CKD progression^13^. A recent study further showed that plant-sourced protein promotes a slower decline in kidney function than animal protein^19^. In our study, patients with advanced CKD were more likely to consume insufficient quantities of vegetables and fruits. These results suggest that CKD patients may require a richer plant-based diet to prevent CKD progression and improve mortality.

Beta carotene has the beneficial effects of an antioxidant^10^. Retinoic acid, which is derived from β carotene, has multiple functions in development, cell differentiation and proliferation, and inflammation regulation. Retinoic acid is known to have reno-protective effects in kidney diseases such as diabetic nephropathy, renal fibrosis, and podocyte injury^11, 12, 20^. We calculated the amount of β carotene intake in CKD patients. Moreover, higher fiber intake improves the gut microbiota and contributes to reducing serum urea and creatinine levels in CKD patients^8^. In this study, the intake of β carotene and dietary fiber was insufficient in advanced CKD patients; the lack of these nutrients may exacerbate CKD. Therefore, a prospective study is needed to elucidate the renal-protective effects of these nutrients.

In this study, the consumption of vegetables and fruits increased in the CKD stage G2 and G3a groups compared to CKD stage G1 (Tables 2, 3, 4). We used the estimated glomerular filtration rate (eGFR) based on serum creatinine for the evaluation of kidney function. However, the production of creatinine is influenced by the amount of body muscle. A previous report indicated that creatinine kinetics are useful in estimating body muscle mass in patients with CKD^21^. Hemodialysis patients with a higher creatinine index have a large amount of skeletal muscle mass^22^. Patients with large amounts of skeletal muscle mass may eat more fruits and vegetables. Therefore, we speculate that patients in CKD stages G2 or G3a tend to eat more fruits and vegetables compared with patients in CKD stage G1. Further investigation is required to support this hypothesis.

The present study has several limitations. First, we used a food frequency questionnaire of 138 food and beverage items from the Japan Public Health Center-based Prospective Cohort Study. The validation studies about this questionnaire indicate moderate correlations between many foods and nutrients intakes and the consumption of various nutrients and foods including vegetables and fruits^23, 24^. However, vegetables and fruits may have been misclassified. Second, dietary information was based on a single assessment; therefore, misclassification was possibly included. Third, given the cross-sectional design of this study, we were unable to describe the causality between the intake of nutrition and kidney function. Fourth, we did not measure the amount of β carotene and dietary fiber from food other than fruits and vegetables. Fifth, fruit consumption in this study was higher than that in healthy adults from the report of the Japanese Ministry of Health, Labour and Welfare, and was therefore likely overestimated. Nevertheless, the information gained in this study contributes to a better understanding of the lack of nutrition in CKD patients.

In conclusion, CKD patients have insufficient vegetable and fruit intake and lack sufficient β carotene and dietary fiber from vegetables and fruits; further investigation is required to determine the association between CKD progression and the lack of this nutrition.

## Methods

### Study design and population

The Fukuoka Kidney Disease Registry is a prospective, multicenter cohort study of non-dialysis-dependent patients with CKD^25^. The inclusion criteria were patients aged ≥ 16 years and patients managed by nephrologists in 12 hospitals in the Fukuoka Prefecture of Japan. CKD was defined in accordance with Kidney Disease: Improving Global Outcomes (KDIGO) criteria. Patients were enrolled between January 2013 to March 2017. The study sample consisted of 4476 outpatients and all patients were scheduled to be followed up for 5 years. Of the 4476 patients, 931 were excluded because of a lack of baseline or food survey data; therefore, data from 3545 patients were analyzed in the present study.

### Clinical parameters

Baseline demographic and clinical data were recorded at enrollment. We obtained the age, sex, height, body weight, and blood pressure of all participants. Clinical biochemical parameters (urinary protein and creatinine, serum albumin, LDL cholesterol, and C-reactive protein concentrations) were determined in a central laboratory. Information regarding each participant’s health, the presence of diabetes, CVD history, and any treatment was collected by clinical research coordinators in our research network from patient medical records using a structured data format. Body mass index (BMI) was calculated as body weight (kg) divided by height (m) squared. Patients were not required to fast when supplying blood samples.

### Classification of chronic kidney disease

CKD was defined and classified according to the patient’s eGFR using the KDIGO guidelines^26, 27^. The eGFR was calculated in patients aged < 18 years using the Schwartz formula and in patients aged ≥ 18 years using the following formula: eGFR (mL/min/1.73 m^2^) = 194 × Cr^−1.094^ × age^−0.287^ (× 0.739 for women). Chronic kidney disease was categorized as stages G1 to G5 in accordance with KDIGO guidelines.

### Classification of proteinuria

Proteinuria was classified according to the KDIGO 2012 clinical practice guideline^28^. Proteinuria was evaluated using the urinary protein/creatinine ratio, which was calculated by dividing urinary protein values by urinary creatinine concentrations. Proteinuria was categorized as < 0.15, 0.15–0.49, and ≥ 0.50 g/gCr.

### Dietary assessment

To assess their diets, enrolled participants were provided with self-administered survey questionnaires including pictures of fruits and vegetables. A semi-quantitative food frequency questionnaire was used to identify the usual intake of 138 food and beverage items and was used in the survey sheet of the Japan Public Health Center-based Prospective Cohort Study^29^. Survey questionnaires were completed by participants and family members. The intake frequency of vegetables and fruits was described in nine categories and ranged from rarely (< 1 time per month) to at least 7 times per day. The standard portion size was recorded as 0.5 times, standard, or > 1.5 times. Daily food intake was calculated by multiplying intake frequency by the standard portion and relative size for each food item in the food frequency questionnaire. From a selection of 138 types of food, 36 types of vegetables—including 17 types of green leafy vegetables—and 15 types of fruits were evaluated. Information on the number of nutrients in each fruit and vegetable was obtained from the Standard Tables of Food Composition in Japan (Seventh Revised Edition)^30^. We multiplied the number of nutrients (β carotene and fiber) in each vegetable and fruit by the number of vegetables and fruits to calculate the intake of β carotene and dietary fiber. The validity of the food survey has been assessed in previous Japan Public Health Center-based Prospective Cohort studies^23, 24^.

### Statistical analyses

Data are presented as mean ± standard deviation for normally distributed continuous variables, median and interquartile range for non-normally distributed continuous variables, and percentage for categorical variables. To evaluate trends in continuous and categorical values, we used the Jonckheere–Terpstra and Cochran–Armitage tests, respectively. Multivariate-adjusted trend tests were analyzed using multiple linear regression analysis. Logistic regression analysis was used to calculate age- and sex-adjusted odds ratios, and multivariable-adjusted odds ratios and 95% confidence intervals were used to determine the relationship between CKD stage and the prevalence of low vegetable intake. The multivariate value of the intake of β carotene and dietary fiber in CKD stages was evaluated by the analysis of covariance. Trend tests were analyzed using multiple linear regression analysis. Comparisons between the value in CKD G1 and those in other groups were made with the Bonferroni test. The intake values of β carotene and dietary fiber were transformed to natural log values to improve the slewed distributions of these values when statistical analyses were conducted; they were then transformed into normal values in tables. The multivariable-adjusted model was adjusted for age, sex, the presence of diabetes, CVD history, systolic blood pressure, BMI, serum albumin concentration, LDL cholesterol, log-C-reactive protein, and urinary protein/creatinine ratio. Statistical analyses were conducted using IBM SPSS version 22 (IBM Corp., Armonk, NY, USA) and R version 3.5.2 (http://www.r-project.org). A two-tailed P value of < 0.05 was deemed to indicate statistical significance.

### Ethics approval

The study was approved by the Clinical Research Ethics Committee of the Institutional Review Board of Kyushu University (approval number 469-09) and the ethics committees of all participating institutions; the study was registered in the University Hospital Medical Information Network clinical trials registry (UMIN000007988) and was conducted in accordance with the principles of the Declaration of Helsinki and its amendments.

### Informed consent

Informed consent was obtained from all patients and/or their legal guardians included in the study.

## Data Availability

The dataset used in this study is under the control of the Data Management Committee of Kyushu University and cannot be shared publicly due to it containing patient data. However, the dataset is available to researchers who need to use the data for individual patient-level meta-analyses or validation studies with another independent cohort. The amended protocol will need to be approved by Kyushu University's ethical committee. Requests can be sent to Toshiaki Nakano, M.D., Ph.D., Kyushu University Hospital (nakano.toshiaki.455@m.kyushu-u.ac.jp).
